# Antisense oligonucleotide induced exon skipping and the dystrophin gene transcript: cocktails and chemistries

**DOI:** 10.1186/1471-2199-8-57

**Published:** 2007-07-02

**Authors:** Abbie M Adams, Penny L Harding, Patrick L Iversen, Catherine Coleman, Sue Fletcher, Steve D Wilton

**Affiliations:** 1 Centre for Neuromuscular and Neurological Disorders, University of Western Australia, Perth, Western Australia; 2 AVI BioPharma, 4575 SW Research Way, Corvallis, Oregon, USA

## Abstract

**Background:**

Antisense oligonucleotides (AOs) can interfere with exon recognition and intron removal during pre-mRNA processing, and induce excision of a targeted exon from the mature gene transcript. AOs have been used *in vitro *and *in vivo *to redirect dystrophin pre-mRNA processing in human and animal cells. Targeted exon skipping of selected exons in the dystrophin gene transcript can remove nonsense or frame-shifting mutations that would otherwise have lead to Duchenne Muscular Dystrophy, the most common childhood form of muscle wasting.

**Results:**

Although many dystrophin exons can be excised using a single AO, several exons require two motifs to be masked for efficient or specific exon skipping. Some AOs were inactive when applied individually, yet pronounced exon excision was induced in transfected cells when the AOs were used in select combinations, clearly indicating synergistic rather than cumulative effects on splicing. The necessity for AO cocktails to induce efficient exon removal was observed with 2 different chemistries, 2'-O-methyl modified bases on a phosphorothioate backbone and phosphorodiamidate morpholino oligomers. Similarly, other trends in exon skipping, as a consequence of 2'-O-methyl AO action, such as removal of additional flanking exons or variations in exon skipping efficiency with overlapping AOs, were also seen when the corresponding sequences were prepared as phosphorodiamidate morpholino oligomers.

**Conclusion:**

The combination of 2 AOs, directed at appropriate motifs in target exons was found to induce very efficient targeted exon skipping during processing of the dystrophin pre-mRNA. This combinatorial effect is clearly synergistic and is not influenced by the chemistry of the AOs used to induce exon excision. A hierarchy in exon skipping efficiency, observed with overlapping AOs composed of 2'-O-methyl modified bases, was also observed when these same sequences were evaluated as phosphorodiamidate morpholino oligomers, indicating design parameters established with one chemistry may be applied to the other.

## Background

Antisense oligonucleotides (AOs) can be used to modify gene expression through the induction of a variety of mechanisms. Oligodeoxyribonucleotides can be used to target a gene transcript for RNaseH induced degradation, whereas oligomers composed of modified bases can redirect gene expression through RNA silencing [1], suppressing specific mRNA translation [2-4], enhancing mRNA stability [5] and redirecting pre-mRNA splicing patterns [6].

Protein-truncating mutations in the dystrophin gene typically lead to Duchenne muscular dystrophy (DMD), the most common severe childhood form of muscle wasting (review, [7]). Although the size of this gene, with 79 exons spanning some 2,400 kb, and distribution of expression have posed major challenges for gene repair or replacement strategies, these features have opened other avenues for intervention, such as AO induced exon skipping. Targeted removal of selected exons can excise or by-pass protein-truncating mutations from the dystrophin pre-mRNA during the splicing process. The application of AOs to induce targeted exon skipping in the dystrophin gene has been reported by several groups, examining different animal models [8-11], regions of the human dystrophin gene transcript [12-14] and a variety of AO chemistries [12,15-18]. We have recently reported a comprehensive list of AOs that can induce skipping of all dystrophin exons, excluding the first and last exons [19]. Many exons could be targeted for excision from the mature dystrophin mRNA with a high level of efficiency and in some cases, two exons were consistently removed using a single AO, suggesting tight coordination of recognition of these exons with intron removal. However, some exons were found to be extremely difficult to dislodge, despite the evaluation of many AOs directed to the target exon. These "recalcitrant" exons could be efficiently excised from the mature mRNA in response to some select combinations of apparently ineffective AOs.

AOs composed of 2'-O-methyl modified bases on a phosphorothioate backbone (2OMeAO) have been used extensively to induce targeted exon skipping in the dystrophin gene transcript and have some advantages over the phosphorodiamidate morpholino oligomers (PMO), including ease and cost of production, and efficient *in vitro *delivery when administered as cationic lipoplexes. However, PMOs appear to be better suited to *in vivo *application, where the increased stability and cellular uptake of uncomplexed compounds allows for higher levels of sustained dystrophin exon skipping, as well as an excellent safety profile [20-22]. In this report, we describe optimization and excision of recalcitrant dystrophin exons from the mature mRNA using AO cocktails for enhanced efficiency and/or specificity. The use of either 2OMeAOs or PMOs does not seem to influence exon skipping trends, indicating optimization of AO design with the 2OMe chemistry should be directly applicable to PMOs.

## Results and discussion

We have designed AOs capable of individually excising 77 of the 79 exons from the dystrophin gene transcript [19], yet no universal motif has been identified as a reliable target for the consistent redirection of dystrophin pre-mRNA splicing. The rationale in our approach to AO design was to first direct AOs at motifs obviously implicated in exon processing and recognition, such as the acceptor and donor splice sites, as well as exonic splicing enhancers as predicted by ESEfinder [23]. Once some dystrophin exon skipping was observed in AO-transfected human myogenic cells, a series of overlapping AOs were then designed to target that area, in an attempt to develop more effective AOs. In many cases, a single AO was eventually developed that would induce substantial levels of targeted exon skipping, and the study would then move to another dystrophin exon. Since this is regarded as a work in progress, the dystrophin exons were regarded as a reference point and classified into four types based upon the ease of excision from the mature mRNA [19]. Type 1 dystrophin exons are removed most efficiently (greater than 30% after *in vitro *transfection at 100 nM), Type 2 are less easily dislodged, while Type 3 exons are poorly excised. Type 4 dystrophin exons are "special cases", where either a single AO removed multiple exons, or multiple AOs are required to excise a single targeted exon.

We observed that approximately two out of three AOs applied to the dystrophin pre-mRNA were able to induce some exon skipping, but there was considerable variation in levels of induced exon removal, relative to the intact dystrophin transcript. In some cases, the exon excision only occurred at low levels, or was sporadic. The frequency of these sporadic exon skipping events was greater than that observed in untreated cells, indicating some interference with the splicing process. However, the lack of reproducibility, or any dose-dependant responses indicated further refinement was essential.

### Dystrophin exon 20 skipping

**Figure 1:**
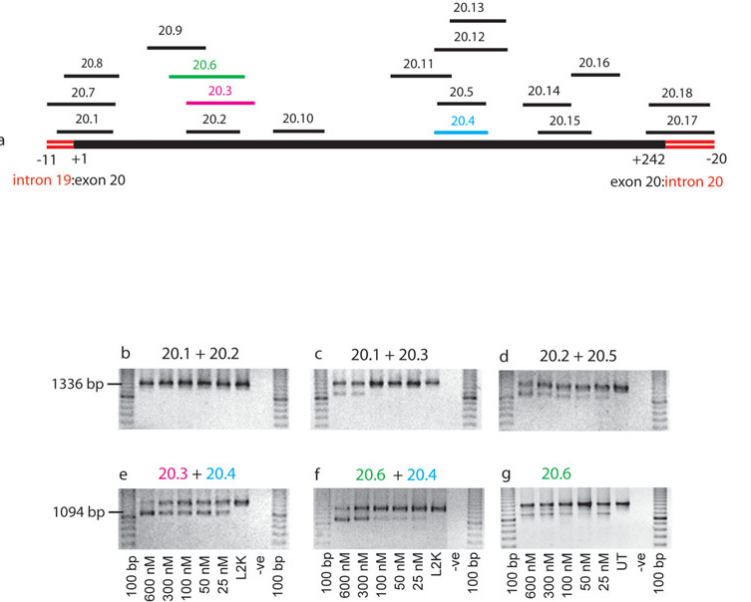
**AO induced excision of human dystrophin exon 20**. Primary human myogenic cultures were transfected with cationic lipoplexes:AO preparations at the concentrations indicated and incubated for 24 hours before total RNA was extracted. Nested RT-PCR was undertaken across exons 18–26. The full-length and exon 20 deleted transcripts are represented by products of 1336 and 1094 bp, respectively. (a) Overview of AO annealing coordinates across dystrophin exon 20. (b) AO cocktail 20.1 and 20.2, (c) AO cocktail 20.1 and 20.3, (d) AO cocktail 20.2 and 20.5, (e) AO cocktail 20.3 and 20.4, (f) AO cocktail 20.5 and 20.6, (g) single AO 20.6.

Dystrophin exon 19 was one example of a Type 1 exon that was very easy to dislodge from the mature mRNA, with every AO directed at acceptor, donor and intra-exonic splicing enhancers able to induce some dystrophin exon skipping [24,25]. In contrast, the following exon in the dystrophin pre-mRNA was one that proved a much greater challenge. Eighteen AOs were prepared to anneal to predicted splice motifs across exon 20 (Additional file 1, Fig 1a), and the majority induced either no, or sporadic exon skipping (data not shown). Several different AO cocktails were then evaluated and found to induce some exon 20 skipping, although consistent variation in efficiency between the different preparations was evident (Figures 1b–f). One preparation, consisting of equal amounts of H20A(+44+71) and H20A(+147+168), was found to be more efficient at exon 20 excision than other combinations and induced the shortened transcript to levels of 36% when compared to the full length product after transfection at 25 nM (Figure 1e). Eventually, a single AO, H20A(+39+69), was developed to induce exon 20 skipping, but this was still not as efficient as the cocktail of H20A(+44+71) and H20A(+147+168) (Figure 1g). Further combinations of AOs were evaluated including AO H20A(+39+69), the most active when used individually, and the non-overlapping AO from the most effective AO cocktail, H20A(+147+168). Unexpectedly, this particular combination was consistently not as efficient at exon 20 excision and only resulted in 13% of the shortened product (Figure 1f), even though there is considerable overlap between the annealing coordinates of H20A(+44+71) and H20A(+39+69). These two AOs targeted essentially the same predicted ESE motifs, with the only differences being H20A(+44+71) extended only one base into a putative SC35 motif, and H20A(+39+69) overlapped one base of a predicted SRp40 motif. Although it seems unlikely that these subtle annealing differences would contribute to the variation in exon excision efficiency, we have shown that the length of an AO is an important parameter in design [34]. While "longer is better" in many instances of single AO-induced exon skipping, this does not hold true in all cases. This presumably arises through the masking of motifs involved in exon silencing or recognition, or influence on secondary structure of the AO. It would appear that combinations of the "best" AOs may not necessarily lead to the optimal AO cocktails, at least for dystrophin exon 20, currently classified as a Type 4 exon.

The same trend in inducing exon 20 skipping was observed in the *mdx *mouse dystrophin gene transcript. Individual AOs were essentially ineffective and one AO combination was most efficient at exon excision [26]. However, it was of interest to note that the AO annealing coordinates of the AOs in the "mouse cocktail" were different from those directed at the human dystrophin gene transcript, indicating that it may not be possible to extrapolate AO design from one species to another. Further comparisons of induced exon skipping between the mouse and human dystrophin gene transcripts are currently underway.

### Dystrophin exon 65 skipping

**Figure 2:**
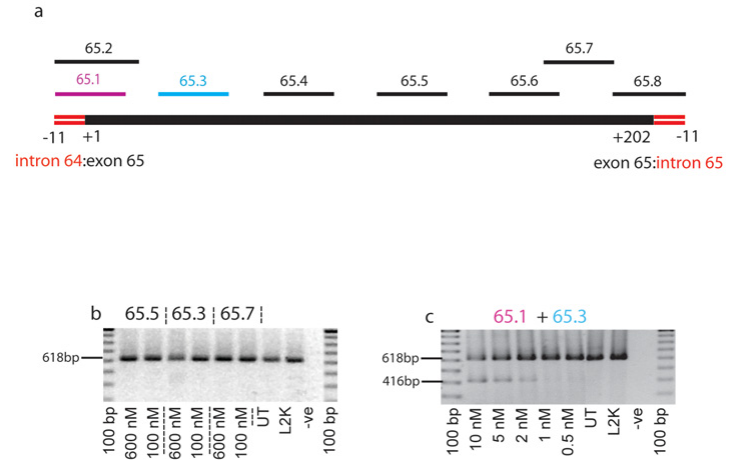
**AO induced excision of human dystrophin exon 65**. Primary human myogenic cultures were transfected with cationic lipoplexes:AO preparations at the concentrations indicated and incubated for 24 hours before total RNA was extracted. Nested RT-PCR was undertaken using primers directed to exons 63–68. The full-length and exon 65 deleted transcripts are represented by products of 618 and 416 bp, respectively. (a) Overview of AO annealing coordinates across dystrophin exon 65, (b) Single AO transfection with 65.5, 65.3 and 65.7, (c) AO cocktail 65.1 and 65.3. Note that the upper concentration of AO:lipoplex administered to these cells was 10 nM in total.

Exon 65 was another dystrophin exon that was difficult to exclude from the mature mRNA. Eight AOs were designed to the donor and acceptor splice sites, as well as putative SR protein binding sites as predicted by ESEfinder [23] (Additional file 2 and Figure 2a). Exon 65 AOs were individually transfected into cultured human myogenic cells as cationic lipoplexes at concentrations of up to 600 nM, but all except one AO failed to induce readily detectable exon 65 skipping (Figure 2b). There was a trace of the shortened transcript missing exon 65, less than 5%, in response to transfection with AO 65.3 (H65A(+26+50)) at 600 nM, but exon 65 excision was not observed at lower concentrations. However, while some AO cocktails were found to be essentially inactive, other combinations were very efficient at inducing exon 65 skipping. The AO cocktail of H65A(-11+14) and H65A(+26+50) was able to induce 65% skipping after *in vitro *transfection at 100 nM (data not shown). Subsequent titrations studies with this AO cocktail indicated 35% exon 65 exclusion after *in vitro *transfection at total concentrations of 10 nM, with 20% skipping at 5 nm and 8% skipping at 2 nM, that is 1 nM of each AO. Approximately 2% exon excision was detected after 0.5 nM transfection, the lowest concentration tested (Figure 2c). Individually, these AOs were unable to induce any exon 65 skipping after transfection at concentrations hundreds of fold greater, again indicating some synergy between these compounds. We originally reported a 3 AO cocktail for exon 65 removal [19], but subsequent studies indicated equivalent efficiency with the combination of only H65A(-11+14) and H65A(+26+50).

### Dystrophin exon 10 skipping

The use of AO cocktails is not limited to enhancing exon removal from the dystrophin mRNA. In the case of human dystrophin exon 10, a combination of 2 AOs was required for specific exon excision. As with other recalcitrant exons, several AOs described in Additional file 3, were unable to induce any detectable removal of exon 10 when applied individually, except for H10A(-05+16), which only excised exon 10 together with blocks of flanking exons (data not shown). Several dystrophin transcripts missing exons 10–12, 9–12, 9–14 and 9–15 were sporadically detected, with 9–12 and 9–14 being most commonly observed. These dystrophin transcripts are in-frame and, extrapolating from a mildly affected Becker muscular dystrophy patient missing exon 9–22 [27], would be expected to produce a shorter dystrophin that could be of near-normal function. Two AOs, H10A(-09+16) and H10A(-05+24) that overlapped H10A(-05+16) had no effect on the processing of the dystrophin transcript (data not shown).

Some AOs directed at other parts of the dystrophin gene transcript had been shown to remove one or two exons in addition to the target, and this was assumed to reflect highly coordinated processing of both exons. Targeting human or canine dystrophin exon 8 always leads to transcripts missing exons 8 and 9 [11,19], whereas directing AOs to human exons 17, 34 or 54 induces transcripts missing the targeted exon as well as 17+18, 34+35 and 54+55 respectively [19].

The induced skipping of exon 10 was distinct from these cases in that larger blocks of exons were involved, and the resultant patterns were somewhat variable. However, upon combining H10A(-06+15) with H10A(+98+119) or H10A(+130+149), specific exon 10 excision could be achieved, although there was still some evidence of additional shortened transcripts induced by the individual AOs.

### Trends in AO cocktail design

Additional file 4 provides an overview of the predicted ESE splice motifs masked by the AOs reported in this study, with an indication of the maximum score and number of motifs shown in brackets. One feature that was common to all effective AO cocktails directed to exon 10, 20 and 65 was the targeting of predicted SC35 motifs by both AOs in the mixture. We do not propose that predicted SC35 motifs are the most important targets for induced exon skipping, as 8 out of 41 AOs targeting Type 1 exons, that are removed at high efficiency, do not appear to be directed at any predicted SC35 motifs [19]. The relevance of the SC35 motifs to induced exon skipping is not known and requires further investigation.

Dystrophin exon 67 had previously been classified as a Type 3 exon, that is, only low levels of exon skipping were induced by the single AO, H67A(+22+47) [19]. This AO was predicted to anneal to 3 SC35 motifs and, while substantial exon skipping was induced after transfection at 600 nM, weaker skipping at 300 nM and there was no detectable skipping at lower concentrations. However, upon combination of H67A(+22+47) with either of two other AOs directed at the acceptor, H67A(-10+17), or donor site H67D(+11-14), 50% exon 67 skipping was generated after transfection at 50 nM, with 42% and 23% exon 67 skipping induced after transfection at 10 and 2 nM, respectively (data not shown). The AOs directed at the exon 67 acceptor and donor sites were shown to be inactive when used individually at concentrations of 600 nM, and neither was directed to predicted SC35 motifs. Although the exon 67 cocktails do not conform to the observation that AOs targeting SC35 motifs are more effective in cocktails, this may reflect on the AO common to both cocktails, H67A(+22+47), which targeted three SC35 motifs and exhibited substantial exon skipping potential when applied at high concentrations.

AO cocktails that induced the most pronounced exon skipping did not necessarily block donor and acceptor sites, and other splicing motifs, nor did the most effective AO combination correlate with the total number of ESE sites targeted. For example, the most effective cocktail for exon 65, H65A(-11+14) and H65A(+26+50) masked three SC55 motifs, a single SF2/ASF and a SRp40 site. In contrast, a less effective AO combination directed at the same exon was directed at 4 SC35 sites, 2 SF2/ASF, 2 SRp40 and a single SRp55 motif. Exons 10 and 65 are removed by AOs directed at the acceptor site and internal ESE's, while exon 20 AOs anneal to internal ESE's. H10A(+98+119) and H65A(+63+87), annealed to all four predicted SR binding sites (SF2/ASF, SC35, SRp40 and SRp55), and these AOs were inactive when used individually.

### AO chemistry comparisons in cocktails

The 2OMeAOs have some advantages over other AO chemistries, including PMOs, in that they can be readily synthesized in-house and may be efficiently transfected into cultured myogenic cells as cationic lipoplexes. PMOs are not readily taken up by cultured cells, unless high transfection concentrations are applied, scrape loading is employed [28,29], or the PMOs are coupled to cell penetrating peptides to enhance delivery [30-32]. We have undertaken other comparisons between the 2OMeAOs and the PMOs directed at the *mdx *mouse nonsense mutation in exon 23 and observed that the PMOs offer much greater potential *in vivo *[16,17,33]. We now extend these studies to other targets, including dystrophin exons that had been difficult to displace and have found the same trends in exon skipping are observed with both chemistries.

**Figure 3:**
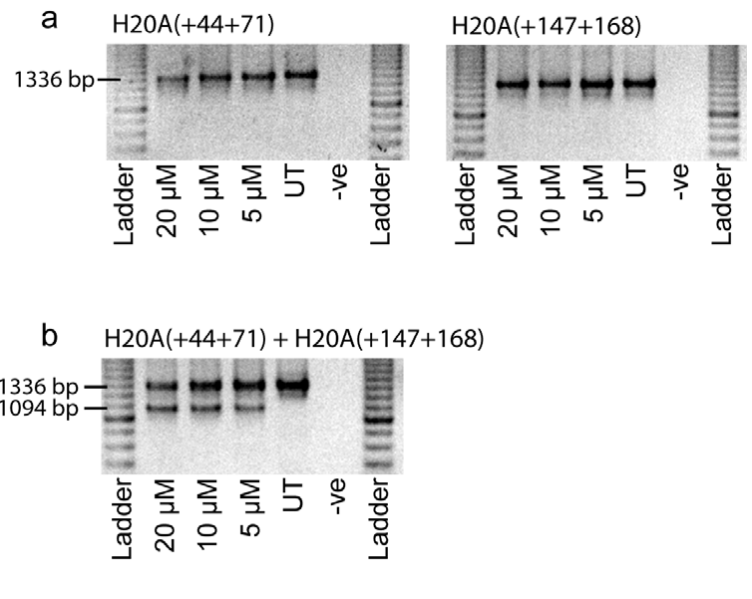
**Induced skipping of human dystrophin exon 20 with PMOs**. Primary human myogenic cultures were transfected with PMOs at the concentrations indicated and incubated for 24 hours before total RNA was extracted. Nested RT-PCR amplified between exons 18 and 26. The full-length and exon 20 deleted transcripts are represented by products of 1336 and 1094 bp, respectively. (a) Individual PMOs directed to exon 20. (b) PMO cocktail directed to exon 20. Note that the total concentration of PMOs is shown, ie, 5 μM indicates 2.5 μM of each PMO.

PMOs directed to the same coordinates as the 2OMeAOs, H20A(+44+71) and H20A(+147+168), were unable to induce any detectable skipping of human exon 20, despite being transfected individually at concentrations of up to 20 μM (Figure 3a and 3b). However, as with the corresponding 2OMeAOs, a combination of the two PMOs resulted in substantial exon 20 skipping after transfection at a total concentration of 5 μM, that is 2.5 μM of each PMO (Figure 3c). These particular PMOs did not carry a peptide tag to enhance delivery, hence the transfection concentration was substantially higher than that used by the 2OMeAO cationic lipoplexes. Subsequent experiments have shown that exon 20 could be excised with the PMO cocktail at total concentrations as low as 1 μM (data not shown), whereas the individual PMOs could not induce skipping at concentrations 40-fold higher.

### Chemistry comparisons in AO design

**Figure 4:**
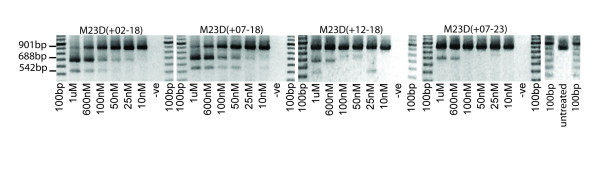
**Induced skipping of mouse dystrophin exon 23 with overlapping PMOs directed at the donor splice site**. Immortalised H2K-mdx myotubes were transfected with PMOs at the concentrations indicated and incubated for 24 hours before total RNA was extracted. Nested RT-PCR was undertaken to amplify exons 20 and 26. The full-length and exon 23 deleted transcripts are represented by products of 901 and 688 bp respectively. The band of 542 bp corresponds to dystrophin gene transcripts missing both exons 22 and 23 and has been previously reported [9, 36].

We have previously reported that the length of an AO can play an important role in the ability of that compound to induce exon skipping [34]. One *mdx *mouse model of muscular dystrophy has a nonsense mutation in exon 23 [35], and has been useful in optimizing AO design [8,9,34,36] and comparing different chemistries [15,17]. We have shown that the donor splice site of mouse dystrophin exon 23 was an amenable target for redirecting splicing and demonstrated a 25 mer, M23D(-7+18) was more efficient than a shorter AO, M23D(+2-18). The latter compound was in turn found to be more efficient at inducing exon 23 excision than 2 longer AOs, M23D(+12-18 and M23D(+7-23) [34]. This same hierarchy of exon 23 skipping was also observed when these sequences were prepared as PMOs, coupled with cell penetrating peptides, and evaluated in cultured cells. RNA was extracted 24 hours after transfection, and as can be seen in Figure 4, the 25 mer, M23D(+7-18), induced superior exon skipping to the 20 mer, which in turn was more effective than both 30 mers, M23D(+12-18) and M23D(+7-23). At later time-points (data not shown), it was possible to establish that PMO M23D(+7-23) was marginally more effective at exon 23 removal than the other 30 mer, confirming the trends shown by the 2OMeAOs [34]. In addition, the shortest products in Figure 4 correspond to dystrophin gene transcripts missing exons 22 and 23, products that are also generated in response to transfection with 2OMeAOs and previously reported [9,36]. Thus trends seen with the 2OMeAOs in terms of ranking of efficiency of overlapping AOs, synergy when applied in cocktails and the more effective compounds also promoting exclusion of exons 22 and 23, correlate to those observed with the PMOs.

All sequences developed as 2OMeAOs and shown to induce skipping of Type 1 exons [19] have now been prepared as PMOs (n = 41). These PMOs were shown to induce the same dystrophin exon removal patterns as those generated by the corresponding 2OMeAOs after transfection in either primary human myogenic cultures or muscle explants (data not shown). Detailed comparisons of sub-optimal PMOs to other dystrophin targets has not been undertaken, as the perfect concordance observed to date between the exon skipping trends with 2OMeAOs and PMOs would suggest that this would be unnecessary and a waste of resources. Our efforts are being directed to fine-tuning the next series of PMOs likely to enter clinical trials, as well as improving upon the efficiency of excision of Type 2 and 3 dystrophin exons, through improved AO design, and/or the use of AO cocktails. It will also be of interest to revisit some of the Type 1 dystrophin exons that are efficiently removed with a single AO, to ascertain if the application of AO cocktails can further enhance exon removal at very low AO concentrations. Although the use of AO cocktails will require the synthesis of two different compounds, these AOs would be used as a single preparation and safety evaluation would be undertaken on the combination. Should an AO cocktail be ten-fold more effective that an optimal single AO to induce targeted exon excision, there would be clear production and cost benefits. Perhaps more importantly, the use of AO cocktails may address safety and efficacy issues in that lower amounts of an AO preparation will need to be administered.

## Conclusion

AO induced exclusion of dystrophin exons during pre-mRNA processing offers a potential treatment for removing or by-passing protein-truncating mutations that lead to DMD. For induced exon skipping to be a viable therapy, the most effective AO preparations must be developed so that minimal amounts can be administered. Many dystrophin exons could be efficiently removed from the mature mRNA by the intervention of a single AO during dystrophin pre-mRNA processing. Some exons require the action of two AOs, often ineffective when used individually, which somehow act in a synergistic fashion, presumably through prevention of spliceosome assembly by altering pre-mRNA folding or masking crucial protein binding sites. The SC35 motif appears to play some role as an amenable target for AO cocktails, but this association is not absolute. Clearly, there are many parameters involved in interfering with the pre-mRNA splicing process remaining to be elucidated. The trends in splice intervention may be seen with AOs composed of 2 different chemistries, 2OMeAO and PMO.

## Methods

### AO design and synthesis

2OMeAOs were prepared on an Expedite 8909 Nucleic acid synthesiser using the 1 μMol thioate synthesis protocol. AOs were designed to anneal to splicing motifs at the intron: exon boundaries, as well as ESE motifs predicted by the web based application, ESEfinder[23].

PMOs, and PMOs conjugated to the cell penetrating peptide, were synthesized by AVI Biopharma (Corvallis, Or). AO nomenclature is based upon that described by Mann et al, 2002 [36]. The first letter designates the species, the number indicates the exon, the second letter specifies Acceptor or Donor splice sites, with the -/+ and numbers representing the annealing coordinates in the intronic and exonic domains respectively. For example, H65A(-11+14) would anneal across the acceptor site of human dystrophin exon 65, specifically to the last 11 bases of intron 64 and the first 14 nucleotides of exon 65.

### Culture and transfection – Primary human myoblasts

The preparation of primary human myoblasts is described by Rando and colleagues, 1994 [37]. Primary human myotubes were transfected in Opti-MEM (Invitrogen), 48 hrs after seeding, with Lipofectamine 2000 (L2K): AO at 1:1 w:w ratio according to manufacturer's instructions (Invitrogen). For each experiment, transfections were repeated three times to confirm reproducibility.

### Culture and transfection – H-2K^b^-tsA58 (H2K) mdx myoblasts

*H2K-Mdx *myoblasts [38] were cultured as described by Mann and colleagues 2001 [9]. AOs were transfected with Lipofectin:AO at 2:1 w:w ratio, 24 hrs after seeding. Lipofectin was used according to manufacturer's instructions (Invitrogen, Melbourne). All transfections occurred in duplicate wells and were repeated three times to ensure consistency.

### Molecular analysis

RNA extraction and RT-PCR have been described previously [8,9]. Briefly, RNA was purified from duplicate cultures using an acid phenol extraction, before a one step RT-PCR was undertaken using specific primers, template and the Invitrogen Superscript III. After 30–35 cycles of amplification, an aliquot was removed and subjected to nested PCR using inner primer sets. Details of all primers used in these experiments are available upon request. The identity of the RT-PCR products was confirmed by direct DNA sequencing [39]. Estimates of relative exon skipping efficiency were performed using the Vilber Lourmat Chemi-Smart 3000 system with Chemi-Capt software for image acquisition and Bio-1D software for image analysis.

## Authors' contributions

AMA and PLH carried out the AO cocktail design and cell transfections, participated in the data acquisition and image preparation. PLI assisted in PMO design and supplied test compounds. CC participated in the AO cocktail evaluation. SF and SDW conceived the study, participated in its design and drafted the manuscript. All authors read and approved the final manuscript.

## Supplementary Material

Additional file 1 **Sequences of AOs designed and evaluated for inducing excision of human dystrophin exon 20**. Reference numbers may be used to orientate the annealing coordinates shown in Figure 1. Click here for file

Additional file 2 **Sequences of AOs designed and evaluated for inducing excision of human dystrophin exon 65**. Reference numbers may be used to orientate the annealing coordinates shown in Figure 2. Click here for file

Additional file 3 Sequences of AOs designed and evaluated for inducing excision of human dystrophin exons 10 and 67. Click here for file

Additional file 4 **Summary of AO combinations evaluated for targeted removal of human dystrophin exons**. Serine-arginine rich protein (SR) binding scores for each AO in the combination are shown. The efficiency of exon removal is indicated (*). (n/a-not applicable). Click here for file

## References

[B1] Braasch DA, Jensen S, Liu Y, Kaur K, Arar K, White MA, Corey DR (2003). RNA interference in mammalian cells by chemically-modified RNA. Biochemistry.

[B2] Amantana A, Iversen PL (2005). Pharmacokinetics and biodistribution of phosphorodiamidate morpholino antisense oligomers. Curr Opin Pharmacol.

[B3] Arora V, Knapp DC, Smith BL, Statdfield ML, Stein DA, Reddy MT, Weller DD, Iversen PL (2000). c-Myc antisense limits rat liver regeneration and indicates role for c-Myc in regulating cytochrome P-450 3A activity. J Pharmacol Exp Ther.

[B4] Deas TS, Binduga-Gajewska I, Tilgner M, Ren P, Stein DA, Moulton HM, Iversen PL, Kauffman EB, Kramer LD, Shi PY (2005). Inhibition of flavivirus infections by antisense oligomers specifically suppressing viral translation and RNA replication. J Virol.

[B5] Vickers TA, Wyatt JR, Burckin T, Bennett CF, Freier SM (2001). Fully modified 2' MOE oligonucleotides redirect polyadenylation. Nucleic Acids Res.

[B6] Wilton SD, Fletcher S (2005). RNA splicing manipulation: strategies to modify gene expression for a variety of therapeutic outcomes. Curr Gene Ther.

[B7] Emery AE (2002). Muscular dystrophy into the new millennium. Neuromuscul Disord.

[B8] Wilton SD, Lloyd F, Carville K, Fletcher S, Honeyman K, Agrawal S, Kole R (1999). Specific removal of the nonsense mutation from the mdx dystrophin mRNA using antisense oligonucleotides. Neuromuscul Disord.

[B9] Mann CJ, Honeyman K, Cheng AJ, Ly T, Lloyd F, Fletcher S, Morgan JE, Partridge TA, Wilton SD (2001). Antisense-induced exon skipping and synthesis of dystrophin in the mdx mouse. Proc Natl Acad Sci USA.

[B10] Bremmer-Bout M, Aartsma-Rus A, de Meijer EJ, Kaman WE, Janson AA, Vossen RH, van Ommen GJ, den Dunnen JT, van Deutekom JC (2004). Targeted exon skipping in transgenic hDMD mice: A model for direct preclinical screening of human-specific antisense oligonucleotides. Mol Ther.

[B11] McClorey G, Moulton HM, Iversen PL, Fletcher S, Wilton SD (2006). Antisense oligonucleotide-induced exon skipping restores dystrophin expression in vitro in a canine model of DMD. Gene Ther.

[B12] Aartsma-Rus A, Kaman WE, Bremmer-Bout M, Janson AA, den Dunnen JT, van Ommen GJ, van Deutekom JC (2004). Comparative analysis of antisense oligonucleotide analogs for targeted DMD exon 46 skipping in muscle cells. Gene Ther.

[B13] Aartsma-Rus A, Janson AA, Kaman WE, Bremmer-Bout M, den Dunnen JT, Baas F, van Ommen GJ, van Deutekom JC (2003). Therapeutic antisense-induced exon skipping in cultured muscle cells from six different DMD patients. Hum Mol Genet.

[B14] Aartsma-Rus A, De Winter CL, Janson AA, Kaman WE, Van Ommen GJ, Den Dunnen JT, Van Deutekom JC (2005). Functional analysis of 114 exon-internal AONs for targeted DMD exon skipping: indication for steric hindrance of SR protein binding sites. Oligonucleotides.

[B15] Gebski BL, Errington SJ, Johnsen RD, Fletcher S, Wilton SD (2005). Terminal antisense oligonucleotide modifications can enhance induced exon skipping. Neuromuscul Disord.

[B16] Gebski BL, Mann CJ, Fletcher S, Wilton SD (2003). Morpholino antisense oligonucleotide induced dystrophin exon 23 skipping in mdx mouse muscle. Hum Mol Genet.

[B17] Fletcher S, Honeyman K, Fall AM, Harding PL, Johnsen RD, Wilton SD (2006). Dystrophin expression in the mdx mouse after localised and systemic administration of a morpholino antisense oligonucleotide. J Gene Med.

[B18] Alter J, Lou F, Rabinowitz A, Yin H, Rosenfeld J, Wilton SD, Partridge TA, Lu QL (2006). Systemic delivery of morpholino oligonucleotide restores dystrophin expression bodywide and improves dystrophic pathology. Nat Med.

[B19] Wilton SD, Fall AM, Harding PL, McClorey G, Coleman C, Fletcher S (2007). Antisense oligonucleotide induced exon skipping across the human dystrophin gene transcript. Molecular Therapy.

[B20] Arora V, Devi GR, Iversen PL (2004). Neutrally charged phosphorodiamidate morpholino antisense oligomers: uptake, efficacy and pharmacokinetics. Curr Pharm Biotechnol.

[B21] Devi GR, Beer TM, Corless CL, Arora V, Weller DL, Iversen PL (4126). In vivo bioavailability and pharmacokinetics of a c-MYC antisense phosphorodiamidate morpholino oligomer, AVI- in solid tumors. Clin Cancer Res.

[B22] Kipshidze N, Tsapenko M, Iversen P, Burger D (2005). Antisense therapy for restenosis following percutaneous coronary intervention. Expert Opin Biol Ther.

[B23] Cartegni L, Wang J, Zhu Z, Zhang MQ, Krainer AR (2003). ESEfinder: A web resource to identify exonic splicing enhancers. Nucleic Acids Res.

[B24] Errington SJ, Mann CJ, Fletcher S, Wilton SD (2003). Target selection for antisense oligonucleotide induced exon skipping in the dystrophin gene. J Gene Med.

[B25] Matsuo M (1996). Duchenne/Becker muscular dystrophy: from molecular diagnosis to gene therapy. Brain Dev.

[B26] Fall AM, Johnsen R, Honeyman K, Iversen P, Fletcher S, Wilton SD (2006). Induction of revertant fibres in the mdx mouse using antisense oligonucleotides. Genet Vaccines Ther.

[B27] Gospe SM, Lazaro RP, Lava NS, Grootscholten PM, Scott MO, Fischbeck KH (1989). Familial X-linked myalgia and cramps: a nonprogressive myopathy associated with a deletion in the dystrophin gene. Neurology.

[B28] Partridge M, Vincent A, Matthews P, Puma J, Stein D, Summerton J (1996). A simple method for delivering morpholino antisense oligos into the cytoplasm of cells. Antisense Nucleic Acid Drug Dev.

[B29] Ghosh C, Iversen PL (2000). Intracellular delivery strategies for antisense phosphorodiamidate morpholino oligomers. Antisense Nucleic Acid Drug Dev.

[B30] Alonso M, Stein DA, Thomann E, Moulton HM, Leong JC, Iversen P, Mourich DV (2005). Inhibition of infectious haematopoietic necrosis virus in cell cultures with peptide-conjugated morpholino oligomers. J Fish Dis.

[B31] Moulton HM, Hase MC, Smith KM, Iversen PL (2003). HIV Tat peptide enhances cellular delivery of antisense morpholino oligomers. Antisense Nucleic Acid Drug Dev.

[B32] Moulton HM, Nelson MH, Hatlevig SA, Reddy MT, Iversen PL (2004). Cellular uptake of antisense morpholino oligomers conjugated to arginine-rich peptides. Bioconjug Chem.

[B33] Fletcher S, Honeyman K, Fall AM, Harding PL, Johnsen R, Steinhaus JP, Moulton HM, Iversen PL, Wilton SD (2007). Morpholino oligomer mediated exon skipping averts the onset of dystrophic pathology in the mdx mouse. Molecular Therapy.

[B34] Harding PL, Fall AM, Honeyman K, Fletcher S, Wilton SD (2006). The influence of antisense oligonucleotide length on dystrophin exon skipping. Mol Ther.

[B35] Sicinski P, Geng Y, Ryder-Cook AS, Barnard EA, Darlison MG, Barnard PJ (1989). The molecular basis of muscular dystrophy in the mdx mouse: a point mutation. Science.

[B36] Mann CJ, Honeyman K, McClorey G, Fletcher S, Wilton SD (2002). Improved antisense oligonucleotide induced exon skipping in the mdx mouse model of muscular dystrophy. J Gene Med.

[B37] Rando TA, Blau HM (1994). Primary mouse myoblast purification, characterization, and transplantation for cell-mediated gene therapy. J Cell Biol.

[B38] Morgan JE, Beauchamp JR, Pagel CN, Peckham M, Ataliotis P, Jat PS, Noble MD, Farmer K, Partridge TA (1994). Myogenic Cell Lines Derived from Transgenic Mice Carrying a Thermolabile T Antigen: A Model System for the Derivation of Tissue-Specific and Mutation-Specific Cell Lines. Developmental Biology.

[B39] Wilton SD, Lim L, Dye D, Laing N (1997). Bandstab: a PCR-based alternative to cloning PCR products. Biotechniques.

